# Effect of self-administered auricular acupressure on smoking cessation --a pilot study

**DOI:** 10.1186/1472-6882-12-11

**Published:** 2012-02-28

**Authors:** Lawrence Leung, Troy Neufeld, Scott Marin

**Affiliations:** 1 Department of Family Medicine, Queen's University, 220 Bagot Street, Kingston, ON K7L 5E9, Canada; 2 Centre of Studies in Primary Care, Queen's University, Kingston, ON K7L 5E9, Canada; 3 Centre of Neurosciences Studies, Queen's University, Kingston, ON K7L 3N6, Canada

## Abstract

**Background:**

Tobacco smoking is still a worldwide health risk. Current pharmacotherapies have at best, a success rate of no more than 50%. Auricular (ear) acupressure has been purported to be beneficial in achieving smoking cessation in some studies, while in others has been deemed insignificant. We hereby describe the protocol for a three-arm randomised controlled trial to examine the possible benefits of self-administered acupressure for smoking cessation.

**Methods:**

Sixty consenting participants with confirmed habit of tobacco smoking will be recruited and randomized into three arms to receive either auricular acupressure at five true acupoints (NADA protocol), auricular acupressure at five sham points, or no auricular acupressure at all. Participants having auricular acupressure will exert firm pressure to each acupoint bilaterally via the bead in the attached plasters whenever they feel the urge to smoke. The treatment phase will last for six weeks during which all participants will be assessed weekly to review their smoking log, state of abstinence, end-exhalation carbon monoxide levels and possible adverse effects including withdrawal reactions and stress levels. At any time, a successful quit date will be defined with continuous abstinence for the following consecutive 7 days. From then on, participants will be evaluated individually for continuous abstinence rate (CAR), end-exhalation carbon monoxide levels and adverse effects of stress and withdrawal at specified intervals up to 26 weeks. Expectancy of treatment will be assessed with a four-item Borkovec and Nau self-assessment credibility scale during and after intervention.

**Discussion:**

We incorporate validated outcome measures of smoking cessation into our randomised controlled trial design with the objectives to evaluate the feasibility and possible benefits of self-administered auricular acupressure as a non-invasive alternative to pharmacotherapy for smoking cessation.

**Trial Registration:**

ClinicalTrials.gov: NCT01389622 (registered Jul 7 2011)

## Background

### Problem of tobacco smoking

Despite a decreasing trend in some countries, tobacco smoking is still prevalent in about one third of our world's population [1] and it remains a major modifiable health hazard with causative links to increased cardiovascular and pulmonary morbidity. It is also associated with lung cancer, oral cancer, laryngeal carcinoma, esophageal carcinoma, hepatocellular carcinoma, pancreatic carcinoma, bladder cancer and cervical carcinoma[2,3]. In Southeast Asia region, there has been a disturbing increase in adolescent smoking[4]. In 2006, it has been estimated that the annual death toll due to tobacco smoking approached 5 million, and is expected to be doubled by 2030[5]. Unfortunately, success of tobacco control is very much predicated by political constraints and the varying efficacies of smoking cessation strategies in any given locality.

### Methods of smoking cessation

Various methods of smoking cessation are available and can be broadly divided into pharmacological and non-pharmacological methods, which often work better in combination[6]. Nicotine replacement therapy is one of the commonest prescribed treatments which enhances the overall rate of quitting by 50-70%[7] with a stand-alone success rate of 6-20%[8-10]. Newer oral medications include sustained-release bupropion[11] and varenicline[12], which significantly improve smoking abstinence with a higher success rate of 30-50%[11-14]. Thus said, the quote success rates may vary according to the length of continuous abstinence which can range from 3 months to 2 years.

### Auricular acupuncture and smoking cessation

**Figure 1:**
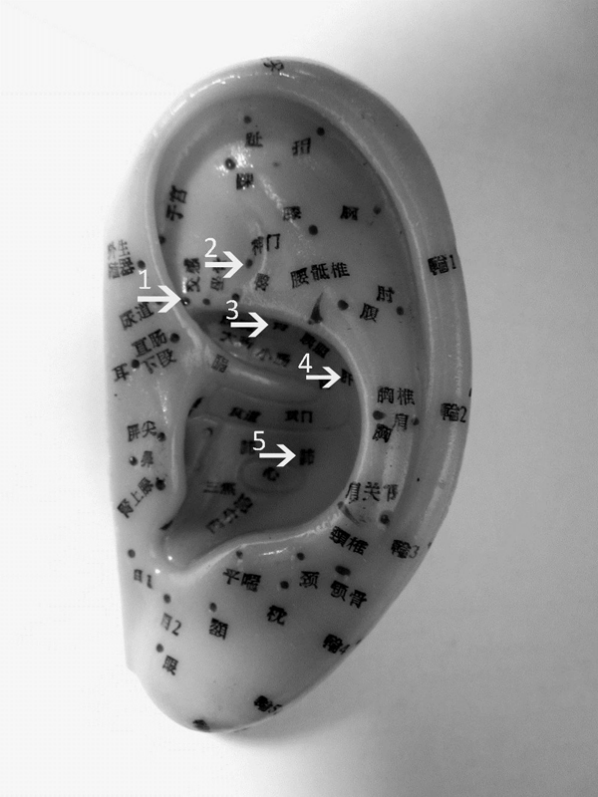
**Auricular points as they are used in acupuncture**. The five auricular points chosen according to the National Acupuncture Detoxification Association (NADA) protocol: Autonomic Point (1); Shen Men (2); C. Kidney (3); Liver (4); Lung 2 (5).

Use of acupuncture for smoking cessation has been practised during the last five decades and its efficacy has been quoted as approaching 45%, which is on par with other pharmacological methods[15]. Auricular (ear) acupuncture is a popular variant with varying degrees of reported efficacy. The practice of auricular acupuncture is based on the theory that there are specific points on the auricle which correspond to major organs or systems of the human body; and they can be manipulated by acupuncture or acupressure to exert a therapeutic effect upon the corresponding target organ or system. An excellent review has been published by Chen in 1993[16]. A recent study shows that electro-stimulation of certain auricular points can modulate vagal activity[17]. A map of commonly used auricular points is included in Figure 1. For smoking cessation, there are various suggested protocols and the 5-points National Acupuncture Detoxification Association (NADA) protocol is the most widely adopted one (5 annotated arrows in Figure 1).

A literature search from all available PubMed listings using the following keywords "Auricular acupuncture" and "smoking" only returns 35 entries with the earliest report of a letter in French in 1975[18]. Since then studies have shown[19-23] conflicting results ranging from statistically significant advantage[23] to not effective as compared to placebo[19,21]. Earlier Cochrane reviews in 2000[24] and 2002[25] both asserted that there was no clear evidence for acupuncture in auricular or non-auricular form for smoking cessation. However, since then clinical data [26-29] has continued to report significant benefits of auricular acupuncture and this led Cochrane Review to amend its position in a meta-analysis conducted in 2006, which concluded that "auricular acupuncture appears to be effective for smoking cessation, but the effect may not depend on point location"[30].

### Auricular acupressure versus needle auricular acupuncture

In the literature, there are two types of auricular acupuncture: with needle inserted into the acupoints or with pressure exerted at the acupoints via plastered beads. Needle auricular acupuncture involves skin penetration and carries the usual risks for acupuncture, i.e., hematoma and infection which may lead to otitis externa[31], auricular chondritis and ear deformity[32] or even subacute bacterial endocarditis[33]. Auricular acupressure via plastered beads is relatively non-invasive and can be self-administered by the recipient at times required. According to the published literature, auricular acupressure has comparable efficacy versus needle auricular acupuncture[30].

To date smoking cessation studies using auricular pressure have been sparse with conflicting results, from no better than sham[34,35] to clinically significant [26,36]. However in those studies, the number and choice of auricular points were not standardised and different types of sham treatments were used. Moreover, the outcome measures (i.e., definition of successful abstinence) were not uniform. In view of this, we design our randomised controlled pilot study to test the hypothesis that self-administered auricular acupressure is a safe and feasible method for smoking cessation, with the aim to calculate the sample and effect size for future randomised double-blind placebo controlled trials.

## Methods/design

### Study aim

To demonstrate that self-administered acupressure at verum auricular acupoints is a safe and feasible option for smoking cessation, and when administered at verum acupoints whether it has benefits as compared to acupressure on non-acupoints and that without acupressure.

### Study design

**Figure 2:**
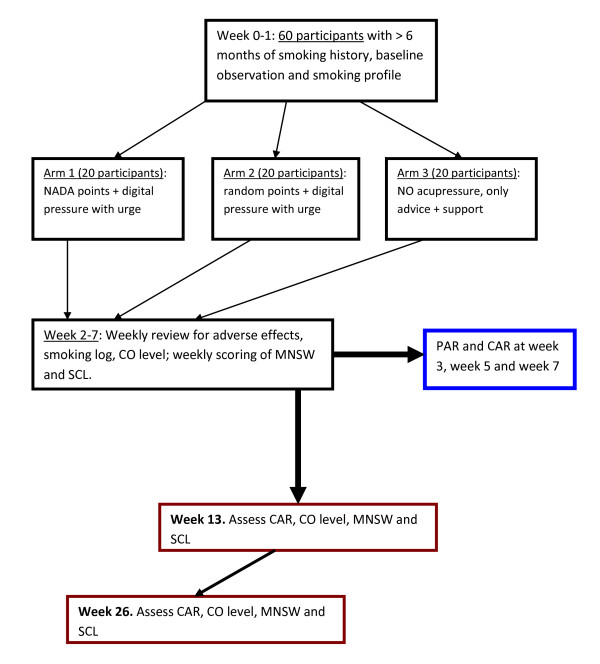
**Flow chart of proposed pilot study**.

This is a pilot study designed as a 3-arm randomised controlled trial. Eligible participants will be recruited from Queen's University Family Health Team (QUFHT) out-patient clinics as per inclusion and exclusion criteria. They will be randomly allocated to self-administered auricular acupressure at true points, self-administered auricular acupressure at sham points, or no auricular acupressure at all. Figure 2 provides an outline of the study.

### Recruitment

Recruitment of participants will be based at the out-patient clinics of the Queen's University Family Health Team (QUFHT) in Kingston, Ontario, Canada. Information pamphlet will be placed in the clinics' waiting areas, supplemented by posters and appropriate referral from physicians in the QUFHT. Respondents will be contacted by research assistants from the Centre of Studies in Primary Care (CSPC) and then invited to an initial screening visit for eligibility.

### Inclusion criteria

Participants are considered eligible if they satisfy the following criteria:

• Male or female, between 18 and 75 years of age inclusive, and able to provide informed consent

• History of tobacco cigarette smoking for at least 6 months.

• Confirmed intention of smoking cessation with no more than 2 previous failed attempts in the last 3 years

### Exclusion criteria

Participants will be excluded if they have:

• Doubts about smoking cessation

• Existing pharmacotherapy for smoking cessation

• History of major psychiatric disorder or chronic pain syndromes

• More than 2 failed attempts of smoking cessation in the previous 3 years, either with pharmacological methods or any forms of acupuncture/acupressure.

• History of substance abuse

• History of atopy, or suspected/known allergy to plaster

### Sample size calculation and randomisation

A convenience sample size of a total of 60 participants will be recruited, with 20 participants per arm after randomisation. Compared to other pilot studies, this sample size is deemed sufficient and appropriate statistical methods will be adopted to accommodate for foreseeable limitations during final analysis (see section on statistical analysis). Randomisation and allocation of subjects will be performed according to the CONSORT standards in terms of randomisation type[37], randomisation generation[38], allocation concealment[39]. All participants will be informed that they will be randomly allocated to one of the three study arms using a computer generated sequence created by the SPSS 11.0 (IBM^© ^SPSS^© ^Statistics, NY, USA). The random allocation will only be disclosed on the date of treatment commencement. Apart from Arm 3 (passive controls with no acupressure), blinding will be ensured in participants of Arm 1 (true acupuncture points) and Arm 2 (sham points). In particular, participants receiving acupressure will be told that the actual points chosen may vary over time to eliminate guessing amongst them during the study. Efforts will also be made to avoid contacts between all participants.

### Auricular acupressure

Auricular acupressure will be administered by research assistants and staff nurses who will attend a one-day training course. They will be naïve to the differences between the true and sham acupressure points, and will be advised not to discuss anything with the participants. Their competence and point accuracy will be assessed before the start of the study and periodically thereafter by the research team and two licenced practitioners in acupuncture.

### Verum acupressure (arm 1)

**Figure 3:**
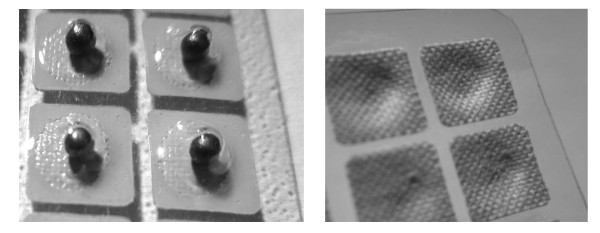
**Figure 3a, b**. Auricular acupressure plasters using *Melastoma candidum *seeds as beads attached under plasters which are individually packaged.

Participants in Arm 1 will receive a 2 mm × 2 mm acupressure plaster (each with a single *Melastoma candidum *seed, see Figure 3a and 3b) at each of the 5 auricular acupoints bilaterally (Lung 2, Shen Men, Autonomic Point, Liver and C. Kidney) according to the NADA protocol (Figure 1). Participants will be instructed to exert firm digital pressure to each acupoint for five seconds via the central plastered seeds whenever they feel the urge to smoke. The seeded plasters are water resistant and should remain in position until the next weekly scheduled review, when they will be replaced with new ones. If the plasters detach, the participants will return to the research center for replacement without delay. Period of intervention will be six weeks (week 2 to week 7).

### Sham acupressure (Arm2)

As active controls, participants in Arm 2 will receive identical acupressure plasters bilaterally as in Arm 1 except that the 5 auricular points are randomly chosen with none belonging to the NADA points, or any other true acupressure points that have been described. If in doubt, the principal investigators will be consulted. As in Arm 1, participants in Arm 2 will also be instructed to exert firm digital pressure to every acupoint via the plastered seeds for five seconds whenever they feel the urge to smoke. Similarly, if the plasters detach at any time of the study, they need to be replaced without delay. Period of treatment will be six weeks (week 2 to week 7).

### Control arm (Arm3)

Participants in Arm 3 will serve as passive controls and no acupressure plasters will be administered. For six weeks (week 2 to week 7) they will be given advice and counselling support for smoking cessation during the weekly review, and will be assessed for outcome measures as with the participants in the other two study arms.

### Co-existent treatment

A record of co-existent treatments, both pharmacological and non-pharmacological, will be recorded for every participant. Any treatment pertaining to smoking cessation should be discontinued at the start of the run-in week and will not be resumed until the participant completed the study or is withdrawn from the study due to treatment failure or adverse events. Equally, all participants will be advised not to attempt any smoking cessation 2 weeks before the run-in period.

### Baseline evaluation

Once eligibility is confirmed, all participants will undergo a pre-treatment baseline evaluation during week 1 (run-in period). All subjects will be subjected to a brief physical examination recording the weight, height, body-mass-index (BMI), waist circumference, resting blood pressure, resting heart rate and end-expiratory carbon monoxide level (CO) through the use of a hand-held Smoklyzer^©^. In addition, a brief history will be taken regarding their past and present health. Their smoking profile will also be assessed in terms of number of cigarettes smoked per day, smoking history in terms of pack-years, number of previous attempts of smoking cessation and methods used, and finally, their level of motivation to quit on Likert scale of 0-7.

### Outcome measures

The primary outcome measures in our study will be 7-day point prevalent abstinence rate (PAR), continuous abstinence rate (CAR), end-expiratory carbon monoxide levels (CO), nicotine withdrawal symptoms according to the Minnesota Nicotine Withdrawal Scale (MNWS) and stress level according to the Stress Check List (SCL). PAR is defined as abstinence from tobacco smoking for the previous 7 days, whereas CAR is defined as abstinence since the target quit date. The time taken (T) to reach the quit date is also recorded. During the active intervention period (week 2 to week 7), the following will be assessed weekly: number of cigarettes smokes per day, CO, MNWS and SCL. At week 3, 5 and 7, CAR and PAR will be assessed. At week 13 and week 26, successful quitters will be assessed for CO, PAR, SCL and MNWS. At week 2 and week 8/one week after successful quitting, expectancy of treatment will be assessed in all participants with a modified 4-item Borkovec and Nau self-assessment credibility scale as adopted by Tough *et al. *[40] in acupuncture related studies.

### Statistical analysis

Baseline characteristics of participants (age, number of cigarettes smoked per day, history of packed-years and number of previous cessation attempts) in all the three arms will be expressed as mean values ± standard deviation (SD). The null hypothesis will be adopted in comparing the outcomes (PAR, CAR, CO levels, MNWS score and SCL scores) between Arm 1 and Arm 3, Arm 2 and Arm 3, and finally between Arm 1 and Arm 2. In view of the small sample size for each arm, the Mann-Whitney-Wilcoxon two-sample rank sum test will be used for each Arm pair. Medians will be calculated with the quartiles and the Mann-Whitney *U*. Where possible, treatment effect for each Arm pair comparison will be expressed using the Hodges-Lehmann estimator with the 95% confidence intervals and significance levels.

### Adverse events and withdrawal from studies

Part of the objectives of our pilot study is to assess the possible adverse effects due either to the auricular acupressure or, due to withdrawal and stress from smoking cessation. The nature and severity of the adverse effects will be recorded and assessed with the MNWS and SCL scores. At any time, participants may withdraw from the study if they develop severe nicotine withdrawal reactions or anxiety symptoms; if they have any adverse effects of pain, discomfort, inflammation or infection at the local sites of the plaster; if there is any inter-current illness that would, in the judgment of the investigators, affect outcome measures to a significant degree; and finally, as requested by the participants.

### Ethics

Consent will be obtained from every participant in a written form. This study protocol has been peer-reviewed and approved by the Queen's University Health Sciences and Affiliated Teaching Hospitals Research Ethics Board. Registration number for ClinicalTrials.gov is.

## Abbreviations

BMI: Body mass index; CAR: Continuous abstinence rate; CO: Carbon monoxide; CSPC: Centre of studies in primary care; MNWS: Minnesota nicotine withdrawal scale; NADA: National acupuncture detoxification association; QUFHT: Queen's university family health team; SCL: Stress Check List; SD: Standard deviation; PAR: Point abstinence rate.

## Competing interests

The authors declare that they have no competing interests.

## Authors' contributions

LL secured funding for the study. LL, TN and CM contributed equally to the design and revision of the research protocol. LL wrote the main manuscript and TN and CM provide critical comments. TN and CM will be principal investigators in the study and LL will be the co-investigator and project supervisor. All authors read and approved the final manuscript.

## Pre-publication history

The pre-publication history for this paper can be accessed here:

http://www.biomedcentral.com/1472-6882/12/11/prepub
